# D-Serine Metabolism and Its Importance in Development of *Dictyostelium discoideum*

**DOI:** 10.3389/fmicb.2018.00784

**Published:** 2018-04-24

**Authors:** Tomokazu Ito, Natsuki Hamauchi, Taisuke Hagi, Naoya Morohashi, Hisashi Hemmi, Yukie G. Sato, Tamao Saito, Tohru Yoshimura

**Affiliations:** ^1^Department of Applied Molecular Biosciences, Graduate School of Bioagricultural Sciences, Nagoya University, Nagoya, Japan; ^2^Department of Materials and Life Sciences, Sophia University, Tokyo, Japan

**Keywords:** D-serine, D-serine dehydratase, serine racemase, D-amino acid oxidase, *Dictyostelium*, development, cAMP

## Abstract

In mammals, D-Ser is synthesized by serine racemase (SR) and degraded by D-amino acid oxidase (DAO). D-Ser acts as an endogenous ligand for *N*-methyl-D-aspartate (NMDA)- and δ2 glutamate receptors, and is involved in brain functions such as learning and memory. Although SR homologs are highly conserved in eukaryotes, little is known about the significance of D-Ser in non-mammals. In contrast to mammals, the slime mold *Dictyostelium discoideum* genome encodes SR, DAO, and additionally D-Ser specific degradation enzyme D-Ser dehydratase (DSD), but not NMDA- and δ2 glutamate receptors. Here, we studied the significances of D-Ser and DSD in *D. discoideum*. Enzymatic assays demonstrated that DSD is 460- and 1,700-fold more active than DAO and SR, respectively, in degrading D-Ser. Moreover, in *dsd*-null cells D-Ser degradation activity is completely abolished. In fact, while in wild-type *D. discoideum* intracellular D-Ser levels were considerably low, *dsd*-null cells accumulated D-Ser. These results indicated that DSD but not DAO is the primary enzyme responsible for D-Ser decomposition in *D. discoideum*. We found that *dsd*-null cells exhibit delay in development and arrest at the early culmination stage. The efficiency of spore formation was considerably reduced in the mutant cells. These phenotypes were further pronounced by exogenous D-Ser but rescued by plasmid-borne expression of *dsd*. qRT-PCR analysis demonstrated that mRNA expression of key genes in the cAMP signaling relay is perturbed in the *dsd* knockout. Our data indicate novel roles for D-Ser and/or DSD in the regulation of cAMP signaling in the development processes of *D. discoideum*.

## Introduction

D-Serine (D-Ser) is highly concentrated in mammalian brain and plays important roles in brain functions such as memory and learning, by binding to *N*-methyl-D-aspartate (NMDA)- and δ2 glutamate receptors (Oliet and Mothet, 2009; Henneberger et al., 2010; Kakegawa et al., 2011). In mammals, D-Ser is endogenously synthesized by serine racemase (SR), a fold-type II pyridoxal 5′-phosphate (PLP)-dependent bifunctional enzyme that catalyzes both Ser racemization and dehydration (Mori and Inoue, 2010; Wolosker and Mori, 2012). SRs are also present in a variety of eukaryotic cells, including plants (Fujitani et al., 2006; Gogami et al., 2009), yeast (Goto et al., 2009), vertebrates (Smith et al., 2010), and invertebrates (Katane et al., 2016; Uda et al., 2016). In tobacco and *Arabidopsis*, SR (and D-Ser) is reported to modulate glutamate-receptor-like channels, which regulate pollen-tube growth and morphogenesis by facilitating Ca^2+^ influx across the plasma membrane and thereby modulating the apical Ca^2+^ gradient (Michard et al., 2011). In mammals, D-Ser is mainly degraded by a flavin-dependent enzyme, D-amino acid oxidase (DAO) (Pollegioni et al., 2007; Konno et al., 2010; Pollegioni and Sacchi, 2010), and D-Ser levels are tightly regulated by the combination of synthesis, degradation, uptake, and/or transport. In accordance with the physiological significance of D-Ser in the regulation of brain functions, altered levels of D-Ser in biological fluids were reported in various neuropsychiatric disorders such as schizophrenia (Bendikov et al., 2007; Cho et al., 2016), Alzheimer’s (Fisher et al., 1998), and ALS (Sasabe et al., 2007).

Although the enzymatic properties of various SRs and DAOs have been reported, the physiological role of SR, DAO, and D-Ser in many eukaryotes except for mammals and plants remains to be elucidated. Interestingly, in addition to DAO, most of the organisms possess additional D-Ser degradation enzyme, PLP- and Zn^2+^-dependent D-Ser dehydratase (DSD). We previously identified and characterized the first example of DSD from *Saccharomyces cerevisiae* (Dsd1p) (Ito et al., 2007, 2008). DSD is highly specific to D-Ser. D-Thr, D-*allo*-Thr, and D-2-amino-3,4-dihydroxybutanoic acid were identified to be poor substrates for DSD (Ito et al., 2007, 2008, 2017). Other proteinogenic L-amino acids and their corresponding enantiomers do not act as the substrates. DSD belongs to the fold-type III PLP-dependent enzymes, and is distinct from classical DSDs such as *Escherichia coli* DsdA, which belong to the fold-type II PLP-dependent enzymes and are independent of Zn^2+^ (Urusova et al., 2012). The wide-range distribution of DSD suggests the importance of this enzyme in many organisms, however, little information is available on its physiological roles. In chicken, DSD is detected in renal cortex, cerebellar cortex, and liver (Nishimura et al., 2014). This localization is similar to that of DAO in mammals. In addition, the chicken DSD is also expressed in cerebrum. On the other hand, DAO activity was not detected in the chicken brain homogenates (Tanaka et al., 2007). Therefore, in chicken brain, DSD but not DAO is considered to be the only enzyme responsible for the degradation of D-Ser and regulates brain function through the control of D-Ser levels.

The cellular slime mold *Dictyostelium discoideum* lives in soil and feeds on bacteria and yeast. When cells are depleted of nutrients, *Dictyostelium* ameba aggregate and undergo multicellular development by releasing the chemoattractant cAMP, ultimately leading to differentiation into fruiting bodies that contain persistent spores (Li and Purugganan, 2010). *Dictyostelium* is used as a model organism for the study of phagocytosis, chemotaxis, cell differentiation, and cell signaling (Devreotes, 1989; Cosson and Soldati, 2008; King and Insall, 2009; Müller-Taubenberger et al., 2013; Loomis, 2014). We found that *Dictyostelium* contains a set of D-Ser metabolic enzymes: SR (Ito et al., 2013b), DAO, and DSD. *D. discoideum* was considered—differently than in mammals—to endogenously synthesize D-Ser by SR and metabolize it by DAO and/or DSD. Interestingly, the *D. discoideum* genome encodes no NMDA receptor or δ2 glutamate receptor, thus suggesting that D-Ser plays different role(s) in the slime mold, mammals, and plants.

In this work, we studied the significance of D-Ser in *D. discoideum*, while focusing on the physiological roles of DSD. Our *in vitro* characterization of the three D-Ser metabolic enzymes (SR, DSD, and DAO) suggests that DSD but not DAO plays a primary role in D-Ser degradation in *D. discoideum*. This was further confirmed by analysis of *dsd*-null *D. discoideum* cells. We found that *dsd*-null cells display delayed early development and impaired efficiency of spore formation. The weak expression of cAMP-signaling genes was identified as a possible cause of the phenotypes. This is the first report that shows the important role of D-Ser and DSD in eukaryotic microorganisms and their implication in the cAMP signaling relay.

## Materials and Methods

### Expression and Purification of DSD and DAO

The cDNAs of *DSD* gene (*DDB_G0289463*) and *DAO* gene (*DDB_G0273783*) were amplified by PCR with KOD-plus ver. 2 DNA polymerase (TOYOBO, Osaka, Japan) using EST clones (DDB0305709 for DSD and DDB0238432 for DAO, from NBRP Nenkin) as a template. Primer pairs used were DSD-pET-fw/DSD-pET-rv (**Supplementary Table S1**) and DAO-pET-fw/DAO-pET-rv (**Supplementary Table S1**). Amplified DNA was purified from agarose gel, subcloned into the pCR-Blunt II-TOPO vector using Zero Blunt TOPO PCR Cloning Kit (Invitrogen, Gaithersburg, MD, United States), and inserted into pET15b (Novagen, Madison, WI, United States). The integrity of the constructed plasmid was confirmed by DNA sequencing. The resultant DSD expression plasmid pET-DdDSD and DAO expression plasmid pET-DdDAO express DSD and DAO, respectively, with an N-terminal 6-histidine tag. *E. coli* Rosetta (DE3) (Novagen) was used as a host cells for the protein expression. The *E. coli* cells were grown at 37°C in Luria-Bertani (LB) medium containing 100 μg/ml ampicillin and 30 μg/ml chloramphenicol. When O.D._600_ reached 0.5, 0.1 mM of isopropyl-β-D-thiogalactopyranoside was added to induce protein expression, and the cells were cultivated for another 14 h at 20°C.

The cells pellet was suspended in Binding buffer (20 mM Tris–HCl, 500 mM NaCl, and 5 mM imidazole, pH 7.9) and sonicated. After centrifugation at 20,000 ×*g* for 30 min at 4°C, and the supernatant was filtered through a 0.45-μm polyethersulfone filter. The filtered lysate was applied to a Ni-NTA His-Bind column (Novagen). The column was washed with Wash buffer (20 mM Tris–HCl, 500 mM NaCl, and 50 mM imidazole, pH 7.9), and eluted with Elution buffer containing 20 mM Tris–HCl, 500 mM NaCl, and 400 mM imidazole (pH 7.9). DSD was concentrated in a buffer consisting of 50 mM sodium phosphate, 300 mM NaCl, 1 mM dithiothreitol, 20 μM PLP, and 20% glycerol (pH 8.0) and stored at -80°C. Ethylenediaminetetraacetic acid (EDTA)-treatment of DSD was performed by dialysis for 16 h at 4°C with a buffer consisting of 50 mM sodium phosphate buffer pH 8.0, 300 mM NaCl, 1 mM dithiothreitol (DTT), 20 μM PLP, 5 mM EDTA, and 15% glycerol. DAO was concentrated with a buffer consisting of 50 mM sodium phosphate (pH 8.0), 300 mM NaCl, 20 μM FAD, and 10% glycerol and stored at -80°C until used.

### Enzyme Assay

Kinetic parameters of DSD were determined as previously described (Ito et al., 2008, 2012a). Reaction mixture (1 ml) contained 50 mM buffer (borate–NaOH buffer, pH 8.5), 20 μM PLP, 0–20 mM D-Ser, 0.3 mM NADH, 10 units of lactic dehydrogenase, and purified DSD. The reaction was carried out at 30°C and the pyruvate released from the substrate was assayed spectrophotometrically at 340 nm.

Enzyme activities of DAO were assayed in a buffer (1 ml) consisting of 50 mM Tris–HCl (pH 8.5), 0.74 mM 4-aminoantipyrine (4-AAP), 0.03% 3-(*N*-ethyl-3-methylanilino)-2-hydroxypropane-sulfonic acid sodium salt (TOOS), 10 units horseradish peroxidase (HRP), 20 μM flavin adenine dinucleotide (FAD), 0–50 mM D-amino acid, and purified DAO. The production of a purple colored product was determined at 550 nm.

### Dictyostelium Culturing

Wild-type (WT) *D. discoideum* AX2 cells and the derivatives were grown axenically in liquid HL5 medium (1.43% Difco Proteose peptone, 0.715% Yeast extract (Nacalai Tesque, Kyoto, Japan), 0.0485% KH_2_PO_4_, 0.0507% Na_2_HPO_4_, and 1.54% glucose, pH 6.5) containing penicillin-streptomycin (×1, Nacalai Tesque) at 22°C according to established protocols as described in dictyBase^1^. When required, blasticidin S and/or G418 sulfate was added to the medium at a final concentration of 10 μg/ml. Cell numbers were counted everyday using a hemocytometer. Alternatively, *D. discoideum* cells were grown on 5LP agar plate (0.5% lactose and 0.5% Difco peptone with 1.5% agar) with *Klebsiella aerogenes* at 22°C.

### Development of *D. discoideum* and Determination of Spore Formation Efficiency

Wild-type and *dsd*-null cells were cultivated axenically in HL5 medium. Cells were harvested in the log phase, washed twice with KK2 buffer (2.2 g/L KH_2_PO_4_ and 0.7 g/L K_2_HPO_4_), and induced to develop synchronously on a nitrocellulose filters (0.45 μm pore size, Black, 47 mm diameter) supported on KK2 buffer-saturated filter papers at a density of 5 × 10^7^ cells/cm^2^. For gene expression and amino acid analyses, developing cells were harvested until 24 h. The cells were washed twice with chilled KK2 buffer and stored at -80°C until used. For the determination of the spore formation efficiency, the cells on the nitrocellulose filters were collected 72 h after initiation of starvation and were resuspended in KK2 buffer containing 0.4% (vol/vol) NP-40. After incubation of 42°C for 45 min, samples were centrifuged at 750 ×*g* for 10 min. The resultant pellet was washed twice with KK2 buffer and resuspended with the same buffer. Spores formed were counted by a hemacytometer.

### Construction of *dsd* Deficient Cells (*Δdsd*)

The *dsd*-null strains (ΔDSD) were produced in AX2 by homologous recombination. The flanking homology regions of *dsd* were amplified by primer pairs Dsd-KO1f/Dsd-KO1r (**Supplementary Table S1**) and Dsd-KO2f/Dsd-KO2r (**Supplementary Table S1**). They were ligated to either side of the blasticidin S resistance gene (*bsr*, from pLPBLP plasmid) using the exogenous restriction sites introduced by the primers during amplification, and cloned into pUC19 plasmid. The disruption cassettes were liberated as *Sph*I/*Kpn*I fragments, and the 10 μg of the fragments were electroporated into *D. discoideum* cells. Transformants were selected at 10 μg/ml of blasticidin S. Three independent *dsd*-null strains were isolated from 96-well plates in HL5 medium containing 10 μg/ml blasticidin S and used for experiments. Disruption of *dsd* was confirmed by PCR using primers pair, Dsd-KOckf/Dsd-KOckr (**Supplementary Table S1**), located outside the disruption cassette.

### Ser Degradation Activity in Cell-Free Extract

Wild-type, ΔDSD, or ΔDSD/*dsd*^+^ were cultivated in HL5 medium. Cells were collected when the cell densities were reached approximately 4 × 10^6^ cells/ml and washed twice with KK2 buffer. Cells were disrupted in a five volumes of a buffer consisting of 50 mM sodium phosphate buffer (pH 7.5), 20% glycerol, and 1× protease inhibitor solution (Nacalai Tesque). After centrifugation at 20,000 ×*g*, 4°C, 20 min, resultant supernatant (cell-free extract) was used for analyses. Protein concentration was determined using the Bradford protein assay (Bio-Rad, Hercules, CA, United States) using bovine serum albumin as a standard. D-Ser degradation activity was determined in a buffer (500 μl) consisting of 50 mM potassium phosphate buffer (pH 8.0), 1 mM D-Ser, 1× protease inhibitor cocktail (EDTA-free, 1:100 dilution, Nacalai Tesque, Kyoto, Japan), and 150 μl of the cell-free extract. L-Ser degradation activity was assayed in a buffer (500 μl) consisting of 50 mM potassium phosphate buffer (pH 8.0), 1 mM L-Ser, 1× protease inhibitor cocktail, 20 μM PLP, 0.1 mM MgCl_2_, 0.1 mM ATP and 150 μl of the cell-free extract. Samples were incubated at 22°C and reaction was terminated by boiling. Ser concentrations in the reaction mixture were quantified by an enzymatic Ser determination assay method (LDH-coupling method) as previously described (Ito et al., 2007). The average rates of D-Ser and L-Ser decomposition during 7.5 h were calculated.

### Amino Acid Analysis

Concentration of D-Ser, L-Ser, and other amino acids were determined by HPLC as described by Ito et al. (2016). Amino acids extracts of the *D. discoideum* cells were prepared by a similar method as described previously (Ito et al., 2013a). The 25 μl of the amino acid extract was mixed to the same amount of the OPA/NAC/Borate reagent [1:9 mix of a NAC/OPA reagent and a 0.4 M borate buffer (pH 9.5)] and incubated for 15 min at 4°C. After the addition of 200 μl distilled water, samples were centrifuged, and the 15 μl-aliquot was applied to the HPLC. The NAC/OPA reagent is a mixture of 10 mg of *N*-acetyl-L-cysteine (NAC) and 20 mg of *o*-phthalaldehyde (OPA) in 1 ml methanol. A 15–41.2% of linear gradient of Buffer B (10 mM Na_2_HPO_4_, 60% methanol, pH 6.5) in Buffer A (10 mM Na_2_HPO_4_, pH 6.5) in 35 min was used for the separation of the diastereomers of amino acid. The flow rate was 0.8 ml/min and the column temperature (Mightysil RP-18 GP II 4.6 × 250 mm ID, 5 μm, Kanto chemical) was kept at 25°C. Excitation and emission wavelengths were 350 and 450 nm, respectively.

### Construction of *dsd*-Complementation Strains

The *dsd* complementary strain, *dsd*-null cells overexpressing *dsd* (ΔDSD/*dsd*^+^), were produced as follows. The cDNA of *dsd* was amplified using PCR with primer pair, Dsd-compf/Dsd-compr (**Supplementary Table S1**). This PCR product was digested with *EcoR*I and *Hind*III and ligated into pDEX-RH (Faix et al., 1992) digested with same restriction enzymes. The resultant plasmid, pDEX-*dsd* was introduced into the *dsd*-null cells by electroporation and selected with 10 μg/ml G418. The pDEX-RH was also introduced into WT and *dsd*-null cells and used as controls.

### Gene Expression Analysis by qRT-PCR

Total RNA was isolated from growing cells and from developing cells using Trizol reagent (Invitrogen) and transcribed using ReverTra Ace (TOYOBO) with oligo(dT) primers, according to the manufacturer’s instructions. Specific primer sets for qRT-PCR were used for amplifying of each gene (**Supplementary Table S1**). qPCR was carried out with ABI StepOne real-time PCR system (Applied Biosystems) and Thunderbird SYBR qPCR Mix (TOYOBO). The qRT-PCR program was as follows: One cycle of 1 min at 95°C followed by 40 cycles of 15 s at 95°C, 30 s at 55°C (58°C for *ig7*), and 1 min at 72°C. Results of the qRT-PCR were analyzed using the comparative CT method (Livak and Schmittgen, 2001). Amplification of *ig7* (mitochondrial large rRNA) was used as a control.

## Results

### Enzymatic Properties of the Two D-Ser Degradation Enzymes DSD and DAO

The *D. discoideum* genome encodes SR (DDB0230209), and unlike mammals two putative D-Ser degradation enzymes: DAO (DDB0238432), and DSD (DDB0305709). The DDB0238432 protein exhibit 33 and 32% sequence identity with human DAO and D-aspartate oxidase, respectively. The DDB0305709 protein share 21.5% sequence identity with DSD of *S. cerevisiae* (Dsd1p). To obtain insight into the significance of D-Ser and its putative metabolic enzymes in *D. discoideum*, we analyzed the enzymes’ properties.

**FIGURE 1:**
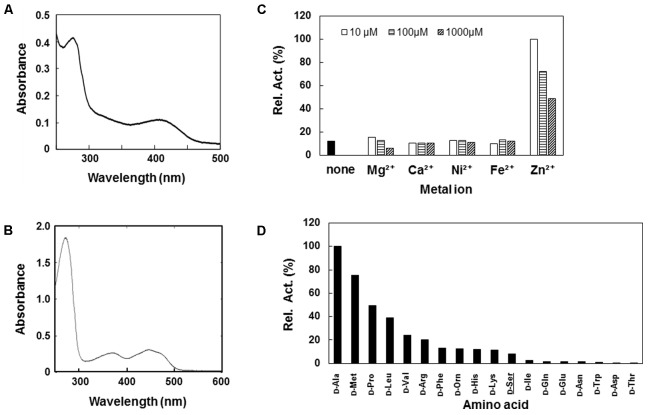
Characterization of recombinant D-Ser dehydratase (DSD) and D-amino acid oxidase (DAO) of *Dictyostelium discoideum*. **(A,B)** UV-vis spectra of purified DSD **(A)** and DAO **(B)**. **(C)** Relative activities of Ethylenediaminetetraacetic acid (EDTA)-treated DSD in the presence of various metal ions. Activity of EDTA-treated DSD was assayed in the presence of 10 mM D-Ser and various concentrations (10μ, 100,μ and 1000 μM) of metal ion. Data are represented as the average of the duplicate measurements. **(D)** Relative activity of deamination activity of DAO with various D-amino acids. Purified DAO was incubated with 10 mM of D-amino acid and the resultant H_2_O_2_ was quantified in the presence of horseradish peroxidase (HRP), TOOS, and 4-aminoantipyrine (4-AAP) at 550 nm. Data are represented as the average of the duplicate determinations.

The putative DSD (DDB0305709 protein) was expressed in *E. coli* using the pET vector system and purified to homogeneity by Ni-affinity chromatography. The UV-visible spectrum of the DDB0305709 protein displayed an absorbance maximum at 420 nm, which is characteristic of the formation of Schiff-base between PLP and amino acid (**Figure 1A**), indicating PLP-binding ability of the protein. The DDB0305709 protein exhibited D-Ser dehydration activity in an optimal pH of 8.0. The *k*_cat_ and *K*_m_ values for the D-Ser dehydration catalyzed by the enzyme were 2.6 s^-1^ and 0.19 mM, respectively (**Table 1**). D-Thr and β-Cl-D-alanine (a D-Ser analog in which the OH group is replaced with a Cl) are known to be substrates of Dsd1p. The DDB0305709 protein also exhibited reactivity toward D-Thr and β-Cl-D-alanine with a catalytic efficiency of 0.9 and 5.3%, respectively, when compared to that of D-Ser (**Table 1**). As with Dsd1p (Ito et al., 2008) and chicken DSD (Tanaka et al., 2011), the DDB0305709 protein required Zn^2+^ for maximal D-Ser dehydration activity: D-Ser dehydration activity was greatly decreased by the EDTA-treatment, and was restored by adding Zn^2+^ (**Figure 1B**). Addition of other divalent metal ions such as Mg^2+^, Ca^2+^, Mn^2+^, Ni^2+^, and Fe^2+^ to the EDTA-treated enzyme did not restore enzyme activity (**Figure 1B**). These results indicate that the DDB0305709 protein, hereafter DSD, is a PLP- and Zn^2+^-dependent DSD.

**Table 1:** Kinetic parameters of recombinant SR, DSD, and DAO of *Dictyostelium discoideum*.

Enzyme (Reaction)	Substrate	*K*_m_ (mM)	*k*_cat_ (s^-1^)	*k*_cat_/*K*_m_
Serine racemase^∗^				
(Racemase)	L-Ser	30 ± 2.7	1.5 ± 0.1	0.05
	D-Ser	34 ± 5.2	1.2 ± 0.1	0.03
(Dehydrase)	L-Ser	40 ± 2.2	0.97 ± 0.02	0.02
	D-Ser	21 ± 1.8	0.22 ± 0.01	0.01
D-Serine dehydratase	D-Ser	0.19 ± 0.02	2.6 ± 0.04	14
D-Amino acid oxidase	D-Ala	0.78 ± 0.05	0.53 ± 0.01	0.68
	D-Ser	6.1 ± 0.78	0.05 ± 0.002	0.01

*^∗^Data were from reference (Ito et al., 2012b, 2013b); SR, serine racemase; DSD, D-Ser dehydratase; DAO, D-amino acid oxidase.*

We also constructed an expression system for the putative *D. discoideum* DAO (DDB0238432). The purified DDB0238432 protein displayed absorption maxima at around 360 and 450 nm, indicating that it possesses an oxidized form of the flavin cofactor (**Figure 1C**). The DDB0238432 protein deaminated various D-amino acids. Among them, D-Ala was the best substrate: *k*_cat_ and *K*_m_ values for the D-Ala oxidase activity were 0.53 s^-1^ and 0.78 mM, respectively (**Table 1**). Little reactivity was observed for D-Asp and D-Glu (**Figure 1D**). These results demonstrated that DDB0238432 encode functional DAO. D-Ser was not a good substrate for DAO (**Figure 1D**), with *k*_cat_ and *K*_m_ values of 0.05 s^-1^ and 6.1 mM, respectively (**Table 1**).

It has become clear that *D. discoideum* possesses three functional enzymes capable of degrading D-Ser: DSD, DAO, and SR. SR was characterized previously (see Ito et al., 2012b, 2013b; **Table 1**). As judged by *k*_cat_/*K*_m_ values, the D-Ser degradation activity of DSD is several hundred-fold higher than that of the other two enzymes (**Table 1**). These results indicate that, unlike in mammals, which use DAO as the primary D-Ser degradation enzyme, D-Ser is probably degraded by DSD in *D. discoideum*.

### DSD Mutation Abolished D-Ser Degradation Activity in the Cell

To examine the physiological significance of DSD in D-Ser metabolism, we constructed a *dsd*-null mutant (ΔDSD). In the mutant strain, *dsd* was replaced by the blasticidin S resistance gene (**Supplementary Figure S1**). The successful generation of *dsd*-null cells demonstrated that *dsd* is not required for the unicellular growth of *D. discoideum* under the conditions tested.

**FIGURE 2:**
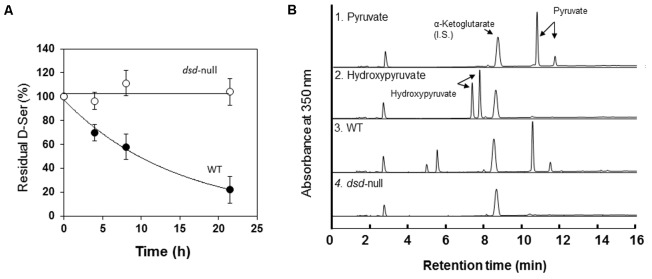
Ser degradation activity of Wild-type (WT) and *dsd*-null strain. **(A)** D-Ser degradation activity in the cell-free extract of WT (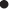) and *dsd*-null cells (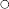). Data are represented as the mean ± SD (*n* = 3). **(B)** Identification of the keto acid product formed from D-Ser by the cell-free extract of WT (3) and *dsd*-null cells (4). Elution profiles of the MBTH-derivatized pyruvate (1) and hydroxypyruvate (2) are also shown as controls. α-Ketoglutarate was used as an internal standard for the derivatization of keto acids as described previously (Ito et al., 2008). Samples after 7.5 h incubation were used for this analysis.

We first determined the D- and L-Ser degradation activities in WT and *dsd*-null cells. D- or L-Ser was incubated with cell-free extract of WT or *dsd*-null cells, and the rates of D- or L-Ser decomposition were quantified. Time-dependent decrease of D-Ser was observed only with the cell-free extract of WT (**Figure 2A**). The average D-Ser decomposition rate during 7.5 h incubation was 15 nmol.h^-1^.mg protein^-1^ with the WT cell-free extract. In contrast, the rate was lower than the detection limit (<0.2 nmol.h^-1^.mg protein^-1^) with the cell-free extract of *dsd*-null cells (**Table 2** and **Figure 2A**). Similar experiments were performed using multicellular-stage cells (cells at 12 h after induction of development). Again, the cell-free extract of WT exhibited D-Ser degradation activity, while that of *dsd*-null cells did not (data not shown).

**Table 2:** Ser degradation activity in the cell-free extract of WT, *dsd*-null, and ΔDSD/*dsd^+^.*

Substrate	WT	*dsd*-null	ΔDSD/*dsd*^+^
L-Ser	10 ± 0.21	12 ± 0.66	11 ± 0.75
D-Ser	15 ± 1.5	N.D.^∗^	312 ± 34

*D- and L-Ser degradation activities in the cell free-extract were analyzed as described in the Section “Materials and Methods.” The average rate of D- and L-Ser degradation during the 4 h incubation was calculated. Data (nmol/h/mg protein) are represented as the mean ± SD (n = 3). ^∗^N.D.: Not Detected; DSD, D-Ser dehydratase; WT, Wild-type.*

In mammals, DAO plays important roles in D-Ser decomposition. Our data suggest that the primary enzyme that degrades D-Ser in *D. discoideum* is DSD. From D-Ser, DSD generates pyruvate, while DAO produces hydroxypyruvate as the keto acid. To examine the contribution of DAO in D-Ser metabolism in *D. discoideum*, the keto acid generated from D-Ser was identified. As shown in **Figure 2B**, with the WT cell-free extract, the keto acid produced from D-Ser was pyruvate. No pyruvate formation was observed with *dsd*-null cells. Hydroxypyruvate were not formed with both WT and *dsd*-null cells (**Figure 2B**). From the above data, we conclude that DSD is responsible for D-Ser degradation in *D. discoideum*.

L-Ser degradation activity in cell-free extracts of WT and *dsd*-null cells was also analyzed. The L-Ser degradation activity was unaffected by the *dsd* mutation (**Table 2**). Little D-Ser was formed from L-Ser, indicating that SR does not play a significant role in L-Ser degradation under the tested conditions.

### Effects of *dsd* Mutation on Growth and Development

**FIGURE 3:**
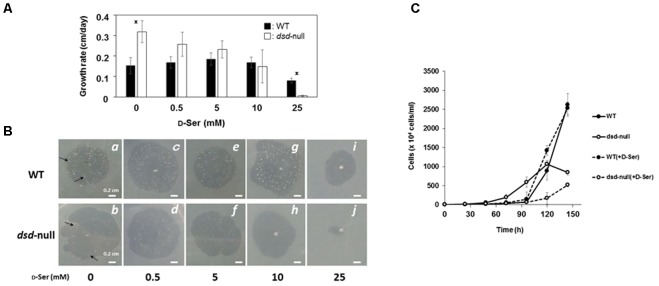
Growth and development of WT and *dsd*-null cells and the effect of exogenous D-Ser. *D. discoideum* cells were grown on *Klebsiella aerogenes* on 5LP agar plates containing indicated D-Ser concentrations. **(A)** The rates of plaque diameter increase of WT (closed bars) and *dsd*-null cells (open bars). Data are represented as the mean ± SD (*n* = 3). Asterisks indicate significant differences (Student’s *t*-test, *P* < 0.05). **(B)** Photographs of plaque of WT (Top) and *dsd*-null cells (Bottom) were taken when the plaque diameter reached approximately 1-cm (4 days in b,d, 5 days in a,c,e–i, 7 days in j). Multicellular structures are indicated as arrows. **(C)** *D. discoideum* cells were grown in HL5 liquid medium in the presence or absence of 10 mM D-Ser. Data are represented as the mean ± SD (*n* = 3).

To examine the effects of the *dsd* mutation on unicellular growth, WT and *dsd*-null cells were grown on 5LP-agar plates along with *Klebsiella aerogenes*, and the size of the plaques formed on the bacterial lawns was measured as an indicator of growth rate. In the absence of D-Ser, the rate of plaque growth was 0.23 cm/day with WT and 0.32 cm/day with *dsd*-null cells, indicating that *dsd*-null cells grew slightly faster than WT (**Figure 3A**). On the 5LP plates, *dsd*-null cells apparently formed fewer multicellular structures (such as slug, mounds, fruiting bodies) than WT did, as judged for both photographs at identical plaque diameters (appeared as dots, see panels *a* and *b* in **Figure 3B**). This indicates that the *dsd* mutation induces delayed initiation of multicellular development.

**FIGURE 4:**
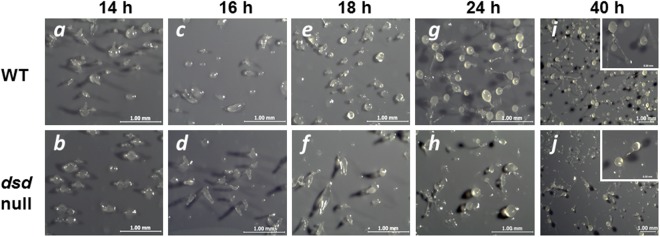
Developmental phenotypes of *dsd*-null mutant. WT (Top) and *dsd*-null cells (Bottom) were developed on the filter membrane at a density of 5.0 × 10^7^ cells/cm^2^. The *dsd*-null cells exhibited aggregation delay and impaired spore formation efficiency compare to WT. Photographs of fruiting body were shown in the inset. Bars indicate 1 mm and 0.5 mm (inset). Experiments were conducted at least twice, and more than three times.

To examine more precisely the effect of the *dsd* mutation on the developmental process, WT and *dsd*-null cells were grown axenically in HL5 medium, whereupon development on nitrocellulose membranes was induced by starvation. At 14 h after induction of development, WT cells were in late mound stage (tip-forming stage), but *dsd*-null cells were in aggregation and/or mound stages (**Figure 4**, panels *a* and *b*). WT cells formed slugs at 16 h (panel *c*) and fruiting bodies at 24 h (panel *g*). In contrast, in *dsd*-null cells, tip-forming aggregates were formed at 18 h development (panel *f*). These observations indicated that *dsd*-null cells show delay in development and apparently spend a prolonged period at the mound stage before proceeding with development. Interestingly, *dsd*-null cells formed fewer fruiting bodies than WT. After 72 h, the number of spores formed in *dsd*-null cells was 6 ± 2% of that in WT.

### Accumulation of D-Ser in *dsd*-Null Cells and Its Effect on Growth and Development

**FIGURE 5:**
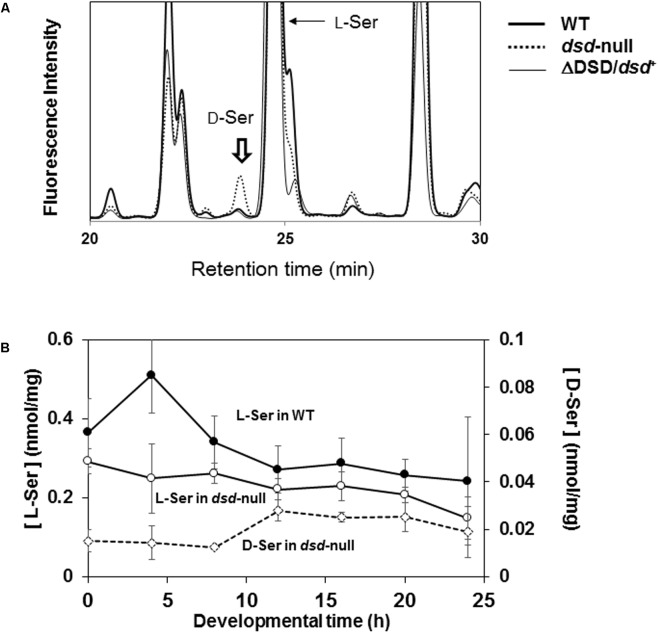
Effect of *dsd* knockout on the intracellular Ser concentration. Intracellular amino acid concentrations of WT, *dsd*-null, and ΔDSD/*dsd*^+^ were determined. **(A)** Representative chromatograms of intracellular amino acid analyses of WT, *dsd*-null, and ΔDSD/*dsd*^+^ of unicellular phase cells. Peak of D-Ser derivative is indicated by arrow. The small peaks at Retention Time 23.5 min in WT and ΔDSD/*dsd*^+^ samples are not for D-Ser derivative (because they were not decreased/disappeared by incubation with Dsd1p). **(B)** Intracellular D-Ser and L-Ser concentrations during development. WT and *dsd*-null cells were grown in HL5 medium and allowed to develop on a filter membrane by starvation. Cells were collected at different stages of development and the intracellular concentrations of D-Ser and L-Ser were quantified by HPLC. Intracellular D-Ser levels were under the detection limit in WT. Data are represented as the mean ± SD (*n* = 3).

Our data suggest that *dsd*-null cells probably accumulate D-Ser, potentially causing the developmental delay and the spore-formation defect. We therefore quantified the intracellular D-Ser levels in WT and *dsd*-null cells (**Figure 5**). D-Ser concentration was under the detection limit in WT cells at the unicellular phase (<0.003 nmol/mg) (**Figure 5** and **Table 3**). In *dsd*-null cells, it was 0.026 nmol/mg, demonstrating the accumulation of D-Ser in *dsd*-null cells. D-Ser concentrations during development were further examined in WT and *dsd*-null cells. D-Ser was not detected throughout development in WT. During the development of *dsd*-null cells, D-Ser was present in the cells at levels of 0.01–0.03 nmol/mg cells (**Figure 5B**). This indicates that D-Ser is likely able to influence all stages of development in the mutant strain.

**Table 3:** Intracellular serine levels of WT, *dsd-null*, and ΔDSD/*dsd^+^*.

	WT	Δ*DSD*	Δ*DSD*/*dsd*^+^
D-Ser	N.D.	0.026 ± 0.018	N. D.
L-Ser	0.98 ± 0.059	0.57 ± 0.12	1.2 ± 0.33

*D. discoideum cells were cultivated in the HL5 medium to a log phase, harvested and washed twice. Amino acids were extracted by a trichloroacetic acid solution, derivatized by NAC and OPA, and separated by a ODS column as described in Section “Materials and Method.” Values (nmol/mg) are the mean ± SD (n = 3). N.D., not detected (<0.003 nmol/mg). DSD, D-Ser dehydratase; WT, Wild-type.*

Our data strongly suggest that the accumulation of D-Ser causes the *dsd*-null phenotypes. To examine whether D-Ser indeed shows inhibitory effects on *D. discoideum* development, cells were grown along with *K. aerogenes* on 5LP plates containing various concentrations of D-Ser; the effect on growth and/or development was then examined. In the presence of 0.5–10 mM of D-Ser, no significant effect on growth or development was observed in WT (**Figure 3**). Delayed development and an inhibitory effect on unicellular growth of WT were observed when 25 mM D-Ser was added to the plates (**Figures 3A,B** panel *i*). In contrast, unicellular growth of *dsd*-null cells was dose-dependently inhibited by D-Ser, and no growth was observed with 25 mM D-Ser (**Figures 3A,B** panel *j*). In all conditions, *dsd*-null cells apparently formed fewer multicellular structures (such as slug, mounds, fruiting bodies) than WT did, as judged when plaque diameters reached 1 cm, and the number of multicellular structures decreased by increasing exogenous D-Ser (*b* > *d* > *f* > *h*, **Figure 3B**). This shows that the developmental delay was further pronounced by exogenous D-Ser in *dsd*-null cells. In addition, the impaired spore-forming ability of *dsd*-null cells was further pronounced by adding exogenous D-Ser. Spore numbers dropped to 1.3 ± 0.3% and 0.3 ± 0.1% of WT in the presence of 1 and 10 mM of D-Ser in the KK2 buffer used for the experiments, respectively. Growth assay was also conducted with the HL5 liquid medium in the presence or absence of 10 mM D-Ser. In the absence of D-Ser, the *dsd*-null cells grew slightly faster than WT in log-phase, but thereafter the increase of cell number was slowed down. The growth of WT was not affected by exogenous D-Ser. In contrast, the growth of *dsd*-null cells was significantly hampered by D-Ser (**Figure 3C**). These observations confirm that D-Ser inhibits and/or delays the unicellular growth, aggregation and spore-forming processes of *D. discoideum*.

### Complementation of *dsd*

**FIGURE 6:**
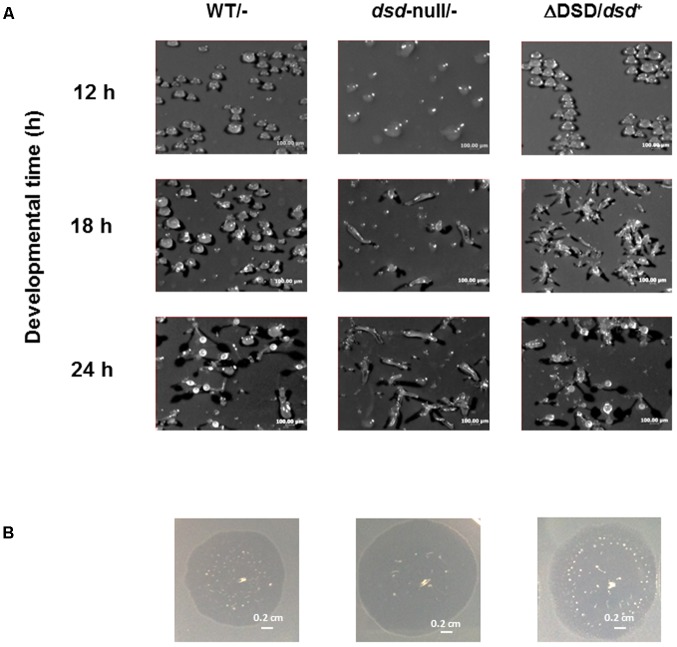
Effect of *dsd* expression in *dsd*-null cells on development. **(A)** WT and *dsd*-null cells both of which harbor empty vector pDEX-RH (WT/- and *dsd*-null/-) and ΔDSD/*dsd*^+^ were developed on filter membrane at a density of 5.0 × 10^7^ cells/cm^2^. Photographs were taken at indicated times. The numbers of spore were counted at 72 h after starvation. The total amount of spore formed in WT was regarded as 100%. **(B)** WT/-, *dsd*-null/-, and ΔDSD/*dsd*^+^ were grown on 5LP plate with *K. aerogenes* at 22°C. Photographs were taken when the plaque diameter reached approximately 1-cm (4 days for WT/-, 3 days for *dsd*-null/-, and ΔDSD/*dsd*^+^). All experiments were performed at least twice, and more than three times.

To demonstrate that the *dsd*-null phenotypes are indeed caused by the *dsd* mutation, we constructed a *dsd* complementary strain (ΔDSD/*dsd*^+^). In this strain, *dsd* was constitutively expressed under the control of the actin15 promoter by using the plasmid pDEX-RH. The cell-free extract of ΔDSD/*dsd*^+^ unicellular ameba exhibited 312 nmol.h^-1^.mg protein^-1^ of D-Ser degradation activity, corresponding to the 20-fold higher expression of *dsd* in ΔDSD/*dsd*^+^ than in WT (**Table 2**). Intracellular amino-acid analysis demonstrated that the ΔDSD/*dsd*^+^ strain does not accumulate D-Ser both in the unicellular (**Figure 5A**) and multicellular phase. On nitrocellulose membranes,ΔDSD/*dsd*^+^ cells developed apparently normally and formed fruiting bodies 24 h after induction of development (**Figure 6A**). Upon *dsd* overexpression, the efficiency of spore formation increased significantly (but not to WT levels). The data suggested that controlled expression of *dsd* is required for the full restoration of the phenotypes of *dsd*-null cells. The number of spores formed in ΔDSD/*dsd*^+^ was 53 ± 10% of that in WT. The delayed initiation of development on 5LP-agar plates with *K. aerogenes* was rescued too (**Figure 6**). These results clearly demonstrate that the phenotypes observed in *dsd*-null cells are caused by the *dsd* mutation.

### *dsd* Mutation Affects the cAMP Signaling Relay

In early development of *D. discoideum*, cAMP plays important roles in intracellular and extracellular signaling (Konijn et al., 1967; Dinauer et al., 1980; Williams et al., 1984; Mahadeo and Parent, 2006). cAMP is required for the chemotaxis of *Dictyostelium* cells and for the activation of the cAMP receptor CarA. CarA is expressed in early development to initiate a signaling cascade for programmed development. The cascade leads to a rapid synthesis of cAMP by the adenylyl cyclase AcaA and to cAMP secretion. This is followed by cAMP degradation mediated by the secreted cAMP phosphodiesterase PsdA (Faure et al., 1990).

**FIGURE 7:**
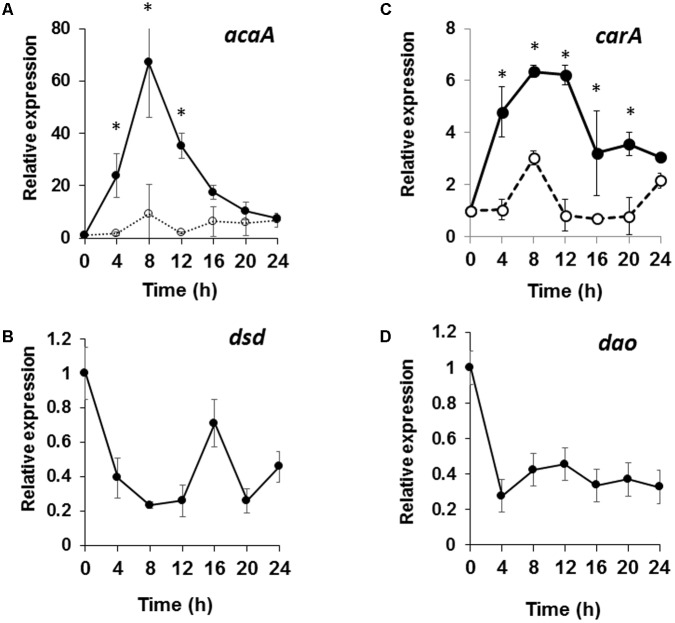
Expression patterns of cAMP signaling genes in *dsd*-null mutant and those of *dsd* and *dao* in WT. WT and *dsd*-null cells growing exponentially in HL5 medium were developed on buffer-saturated filters. Total RNA was extracted at the indicated time points and the expression levels of *acaA* **(A)**, *carA* **(B)**, *dsd* **(C)**, and *dao* **(D)** were quantified by qRT-PCR. The mRNA level of each gene was normalized by the expression of *ig7* mRNA in the respective sample. Values are represented as the mean ± SD (*n* = 3). Asterisks indicate significant differences between WT and *dsd*-null cells (Student’s *t*-test, *P* < 0.05).

We hypothesized that the developmental delay in *dsd*-null cells may be caused by a defect and/or perturbation in the cAMP signaling relay. To examine this, the expression of the key cAMP signaling genes, *carA* and *acaA* was analyzed (**Figures 7A,B**). It is known that, in WT, the mRNA levels of *carA* and *acaA* peak at 4–8 h after starvation (Faure et al., 1990; Narita et al., 2014). Indeed, in WT, the mRNA levels of *acaA* peaked at 8 h and increased 50-fold after starvation (**Figure 7A**). In *dsd*-null cells, expression of *acaA* peaked at 8 h but increased only 17-fold. The same was observed for *carA*. In WT, mRNA levels of *carA* increased sixfold at 8 h, but only threefold in *dsd*-null cells (**Figure 7B**). Although no significant differences were observed, the *pdsA* expression was slightly delayed. The expression of *pdsA* was peaked at 4 h in WT and at 8 h in *dsd*-null cells (data not shown). In addition, we found that *dsd* is expressed constitutively during development, and is most highly expressed in early development (**Figure 7C**). This may reflect the significance of D-Ser regulation particularly in the initiation of development. The decreased expression of the key cAMP-signaling genes during aggregation may serve to explain the delayed aggregation process in *dsd*-null cells. Our data suggest that DSD plays an important role in the regulation of cAMP-signaling genes in early development in *D. discoideum*.

## Discussion

In this work, we studied the physiological significance of D-Ser in *D. discoideum*, focusing on the role of PLP- and Zn^2+^-dependent DSD. Our characterization of the three D-Ser metabolic enzymes (SR, DAO, and DSD) indicates that D-Ser is potentially synthesized by SR and mainly metabolized by DSD in *D. discoideum*. The fact that (i) *D. discoideum* DAO is not a D-Ser-specific enzyme and exhibits low catalytic activity toward D-Ser, (ii) *dsd*-null cells completely lost their D-Ser degradation activity in cell-free extract, (iii) the mutant cells accumulated intracellular D-Ser, (iv) and no hydroxypyruvate formed from D-Ser, all support the conclusion that DAO plays little role in D-Ser degradation in *D. discoideum* under the conditions examined. This situation is different from the one in mammals. There, D-Ser is metabolized by an SR–DAO enzyme system, in which D-Ser is synthesized by SR and mainly degraded by DAO. In WT *D. discoideum*, intracellular D-Ser is kept at considerably low levels (<0.001 nmol/mg cells), and DSD contributes to the maintenance of low levels of intracellular D-Ser. In nature, *D. discoideum* normally eats bacteria and/or yeast on soil surface; the bacterial cell wall contains D-Ala as a component of peptidoglycan. Indeed, *DAO* is most highly expressed in unicellular phase (**Figure 7D**). To our knowledge, *D. discoideum* does not possess any enzyme—such as alanine racemase, D-Ala dehydrogenase, and/or D-amino acid aminotransferase—capable of metabolizing D-Ala, with the exception of DAO. We thus expect that DAO may participate in D-Ala decomposition in the cell.

We found that *dsd*-null cells exhibit delayed development and impaired spore-formation ability. In *dsd*-null cells, these phenotypes were further pronounced by exogenous D-Ser. In addition, high concentration of D-Ser induced delayed development in WT. These data show the importance of maintaining low levels of D-Ser for the normal development of *D. discoideum*. Indeed, *dsd* is expressed constitutively during development, and is most highly expressed in early development. This may reflect the significance of D-Ser regulation particularly in the initiation of development.

The molecular mechanism of the D-Ser-induced inhibition of developmental processes is interesting but is not fully determined. qRT-PCR analysis revealed that one cause of the slow development is the weak expression of the genes responsible for cAMP relay (*acaA* and *carA*) in early development. This is the first report of the involvement of D-Ser and DSD in the cAMP signaling relay during development. It is known that some aminoacyl-tRNA synthases mischarge D-amino acids on tRNAs (Calendar and Berg, 1966; Soutourina et al., 2004). Accumulated D-Ser may be mischarged to inhibit protein synthesis in *D. discoideum*.

We are able to speculate that D-Ser may have negative effects on L-Ser and/or coenzyme A (CoA) biosynthesis. In *E. coli*, D-Ser is known to act as a powerful inhibitor of 3-phosphoglycerate dehydrogenase, the first enzyme in L-Ser biosynthesis, and as a competitor of β-alanine in the synthesis of pantothenate catalyzed by pantoate-β-alanine ligase (Cosloy and McFall, 1973). The *D. discoideum* genome encodes a 3-phosphoglycerate dehydrogenase (DDB_G0281071) and a pantoate-β-alanine ligase (DDB_G0288935). Indeed, we observed slightly decreased intracellular levels of L-Ser in *dsd*-null cells. The decrease in intracellular L-Ser concentration may reflect a weakened L-Ser biosynthetic pathway in the mutant strain.

We may consider the possibility that D-Ser (and DSD) may be involved in the ammonia production in *Dictyostelium*. Ammonia is implicated in a number of key processes in *Dictyostelium* development, including aggregation and culmination (Williams et al., 1984; Riley and Barclay, 1990). Williams et al. reported the importance of ammonia in the modulation of cAMP relay during development in *D. discoideum* (Williams et al., 1984). The primary source and the enzymes responsible for ammonia production in *D. discoideum* have not been identified. DSD knockout could attenuate ammonia production from D-Ser and induce the developmental defects. We are currently studying D-Ser with regard to these possibilities (protein synthesis, L-Ser biosynthesis, CoA biosynthesis, and/or ammonia production), as well as its implication in the cAMP signaling relay in *D. discoideum* early development.

## Conclusion

We studied the significances of D-Ser especially focusing on the enzymes responsible for D-Ser catabolism in *D. discoideum*. Although *D. discoideum* possesses SR and DAO, our data demonstrated that DSD is responsible for D-Ser degradation. By generating DSD deficient *D. discoideum* strain, we showed the importance of DSD in the early development and spore formation efficiency. We proposed novel roles for D-Ser and/or DSD in the regulation of cAMP signaling in the development processes of *D. discoideum*.

## Author Contributions

TI designed the study, performed and analyzed the all experiments, and wrote the paper. NH performed the experiments shown in **Figures 3**–**7**. TH constructed the DSD mutant strain and performed the experiments shown in **Figures 2**, **3**. NM characterized the DAO activity *in vitro* (**Figure 1**). HH discussed and analyzed the data. YS constructed the *dsd*-complementary strain. TS discussed and analyzed the data and wrote the paper. TY designed the study, analyzed the data, and wrote the paper. All authors reviewed the results and approved the final version of the manuscript.

## Conflict of Interest Statement

The authors declare that the research was conducted in the absence of any commercial or financial relationships that could be construed as a potential conflict of interest.

## Footnotes

1 www.dictybase.org

## Supplementary Material

FIGURE S1 Construction of *dsd*-null mutant. **(A)** The blasticidin S resistance gene (*bsr*) was introduced into the Wild-type (WT) strain of *Dictyostelium discoideum* by homologous recombination. **(B)** Agarose gel electrophoresis of PCR-amplified DNA with DsdKOckf (a) and DsdKOckr (b) primers from the parental strain (WT) and the *dsd*-null mutants. The sizes of marker DNA fragments (Quick-Load 1 kB DNA ladder; New England BioLabs) are shown on the left.

TABLE S1 Primers used in this study.
