# Supplementary figures and images for: D-Serine Metabolism and Its Importance in Development of Dictyostelium discoideum
Source: Front Microbiol. 2018 Apr 24;9:784. doi: 10.3389/fmicb.2018.00784 (PMC5928759; doi:10.3389/fmicb.2018.00784)

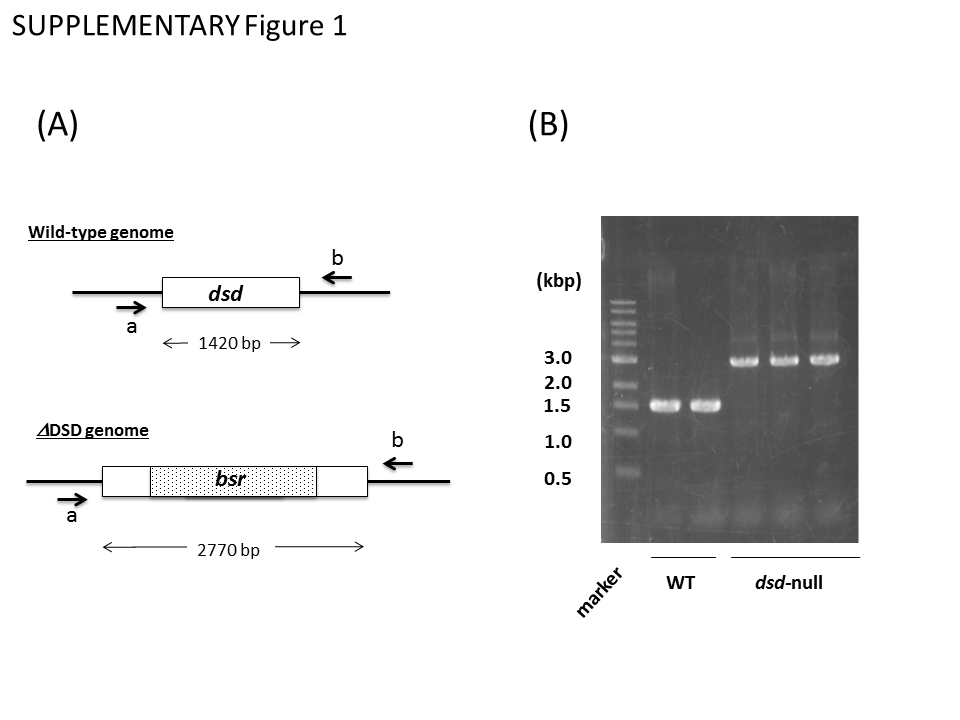

Supplement: FIGURE S1 — Construction of dsd-null mutant. (A) The blasticidin S resistance gene (bsr) was introduced into the Wild-type (WT) strain of Dictyostelium discoideum by homologous recombination. (B) Agarose gel electrophoresis of PCR-amplified DNA with DsdKOckf (a) and DsdKOckr (b) primers from the parental strain (WT) and the dsd-null mutants. The sizes of marker DNA fragments (Quick-Load 1 kB DNA ladder; New England BioLabs) are shown on the left. [file Image_1.TIF]
