# Supplementary material for: D-Serine Metabolism and Its Importance in Development of Dictyostelium discoideum
Source: Front Microbiol. 2018 Apr 24;9:784. doi: 10.3389/fmicb.2018.00784 (PMC5928759; doi:10.3389/fmicb.2018.00784)
Supplement: TABLE S1 — Primers used in this study. [file Table_1.DOCX]

Supplementary Table 1 Primers used in this study

| Primer Name | Sequence (5´ to 3´) | Site for restriction enzyme |
| --- | --- | --- |
| DSD-pET-fw | CCGCTCGAGATGAATGAAAATAATGATGATATTAAT | XhoI |
| DSD-pET-rv | CCCAAGCTTTTACCAATGTTTATTTGGTTTAAATG | HindIII |
| DAO-pET-fw | CCTCCGCTCGAGATGAATCAAAATAAAAATAATAATAATAAAAATC | XhoI |
| DAO-pET-rv | TTATAATTTACTTATTAATTTATTAAAATG |  |
| Dsd-KO1f | AAACTGCAGCATCACTAGGTGTAAATGTTAGAC | PstI |
| Dsd-KO1r | TTGGTACCCCTCCAACCGGTGTTGTAATATC | KpnI |
| Dsd-KO2f | AAGGATCCGGTGTTCATGTATTGGCAACCATAG | BamHI |
| Dsd-KO2r | TTGTCGACCTGTGACTCTAGCTTCCTCAATTG | SalI |
| Dsd-KOckf | CACCATGTGTTTTAGTATTGGATTCAGTTG |  |
| Dsd-KOckr | CCCAGAACCAACAAGTTTTGACCG |  |
| Dsd-compf | ATAGAATTCATGAATGAAAATAATGATGATATTAAT | EcoRI |
| Dsd-compr | TATAAGCTTACCAATGTTTATTTGGTTTAAATG | HindIII |
| qPCR-acaA-fw | ATGCAATCCAATGCTCAAGATAATG |  |
| qPCR-acaA-rv | AATGAGCCAATTTCACCCAAGAG |  |
| qPCR-carA-fw | CCAGCACTCAATATTCTCC |  |
| qPCR-carA-rv | ATGATGATAAAGAAGATGAAGATGAACC |  |
| qPCR-IG7-fw | TTACATTTATTAGACCCGAAACCAAGCG |  |
| qPCR-IG7-rv | TTCCCTTTAGACCTATGGACCTTAGCG |  |
| qPCR-DSD-fw | CAGATCCAAAATTACAARCAACCACTG |  |
| qPCR_DSD-rv | TCCATAATCACCTGTGACTCTAGCTTC |  |
| qPCR_DAO-fw | CTCAAATAAAGCAGCAGCAATTATGG |  |
| qPCR_DAO-rv | AGTGTCCATAACGAAACCGTCATC |  |
